# The selective D_3_Receptor antagonist VK4-116 reverses loss of insight caused by self-administration of cocaine in rats

**DOI:** 10.1038/s41386-024-01858-7

**Published:** 2024-04-06

**Authors:** Marios C. Panayi, Shohan Shetty, Micaela Porod, Lisette Bahena, Zheng-Xiong Xi, Amy Hauck Newman, Geoffrey Schoenbaum

**Affiliations:** National Institute on Drug Abuse Intramural Research Program, 251 Bayview Boulevard, Baltimore, MD 21224 USA

**Keywords:** Neuroscience, Addiction

## Abstract

Chronic psychostimulant use causes long-lasting changes to neural and cognitive function that persist after long periods of abstinence. As cocaine users transition from drug use to abstinence, a parallel transition from hyperactivity to hypoactivity has been found in orbitofrontal-striatal glucose metabolism and striatal D_2_/D_3_-receptor activity. Targeting these changes pharmacologically, using highly selective dopamine D_3_-receptor (D_3_R) antagonists and partial agonists, has shown promise in reducing drug-taking, and attenuating relapse in animal models of cocaine and opioid use disorder. However, much less attention has been paid to treating the loss of insight, operationalized as the inability to infer likely outcomes, associated with chronic psychostimulant use. Here we tested the selective D_3_R antagonist VK4-116 as a treatment for this loss in rats with a prior history of cocaine use. Male and female rats were first trained to self-administer cocaine or a sucrose liquid for 2 weeks. After 4 weeks of abstinence, performance was assessed using a sensory preconditioning (SPC) learning paradigm. Rats were given VK4-116 (15 mg/kg, i.p.) or vehicle 30 min prior to each SPC training session, thus creating four drug-treatment groups: sucrose-vehicle, sucrose-VK4-116, cocaine-vehicle, cocaine-VK4-116. The control groups (sucrose-vehicle, sucrose-VK4-116) showed normal sensory preconditioning, whereas cocaine use (cocaine-vehicle) selectively disrupted responding to the preconditioned cue, an effect that was reversed in the cocaine-VK4-116 group, which demonstrating responding to the preconditioned cue at levels comparable to controls. These preclinical findings demonstrate that highly selective dopamine D_3_R antagonists, particularly VK4-116, can reverse the long-term negative behavioral consequences of cocaine use.

## Introduction

Substance use disorders (SUDs) are characterized by persistent drug use despite negative consequences and repeated intentions to abstain [1, 2]. In drug users, including cocaine use disorder (CUD), this behavioral phenotype may reflect a lack of insight [3–5]. A lack of insight has been operationalized as an inability to mentally simulate and infer the causes and effects of one’s own behavior [6, 7] and is associated with reduced function in orbitofrontal cortex (OFC) networks [3, 8, 9]. Consistent with this, chronic cocaine use in CUD is consistently associated with decreased functional activity of the orbitofrontal cortex (OFC), and in a preclinical model, abstinent rats with a history of cocaine self-administration (SA) are impaired on insight-based behavioral tasks that require integrating learned information in the OFC to make inferences [10, 11]. These deficits are associated with the loss of critical task representations in OFC and can be successfully treated by optogenetic activation of pyramidal neurons in OFC [7]. This suggests that targeting OFC networks is a possible therapeutic pathway to treat the long-term consequences of chronic cocaine use.

Cocaine use has also been associated with increased D_3_R availability in the striatum and midbrain [12–17], an increase which correlates with OFC hypofunction in CUD patients [18–20]. Midbrain D_3_Rs in substantia nigra and ventral tegmental area are thought to be inhibitory modulators of presynaptic dopamine release in striatum and OFC [21–25]. In non-clinical populations, midbrain D_3_R availability is correlated with both resting state OFC activity, and the functional connectivity between OFC subregions and multiple key large-scale neural systems such as salience executive control networks, basal ganglia/limbic network, and the default mode network [26]. Recent evidence also supports a causal link between cocaine use, increased midbrain D_3_R availability, OFC hypofunction, and behavioral deficits in tasks like probabilistic reversal learning [27–29].

Accordingly, D_3_R-antagonists/partial agonists have been shown to successfully reduce both self-administration and relapse to drug-seeking as assessed by reinstatement to drug-seeking caused by psychostimulants such as cocaine and nicotine in rodents [30–33], as well as opioids such as oxycodone and heroin [34–38]. However, until recently, most of the available D_3_R-antagonists have had only moderate (<100-fold) D_3_R/D_2_R selectivity [25]. This limitation has been overcome by the development of class of highly selective D_3_R-antagonists with low D_2_R binding affinity, including the compound VK4-116 [34, 39, 40]. Here we tested whether the novel D_3_R-antagonist VK4-116 could treat the deficits in insight-based behavior that occur following chronic cocaine use in a preclinical rodent model [7, 10, 11].

## Materials and methods

For more specific details regarding subjects, drugs, apparatus, surgery, self-administration, preconditioning, exclusion criteria, and data analysis, see Supplementary Material and Methods Online. All procedures were approved by the NIDA-IRP Animal Care and Use Committee.

The goal of the experiment was to replicate the effect of cocaine self-administration on performance of an orbitofrontal-dependent sensory preconditioning task, shown previously [8, 10], and to assess the effects of pretreatment with VK4-116, a selective D_3_ antagonist, at a dose of 15 mg/kg. This dose was selected as the optimal dose based on previous work showing that it successfully disrupts oxycodone self-administration while leaving oral sucrose self-administration intact in Long-Evans rats [34].

For this, rats were food restricted and then trained to self-administer either cocaine or a food reinforcer for 2 weeks; 4 weeks later they were trained in the 3-phase sensory preconditioning (SPC) task used previously [8, 10], after receiving i.p. injections of either VK4-116 [(±)-N-(4-(4-(3-chloro-5-ethyl-2-methoxyphenyl)piperazin- 1-yl)-3-hydroxybutyl)-1H-indole-2-carboxamide] or vehicle 30 min prior to each session.

**Fig. 1:**
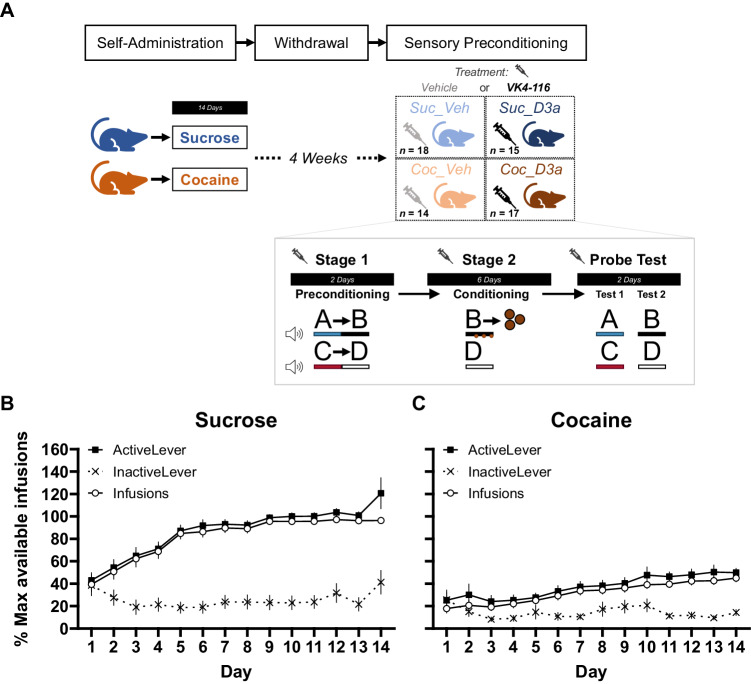
Experimental timeline and self-administration behavior. **A** *Timeline of experimental procedures:* (top) Rats first performed self-administration training (SA), followed by 4 weeks of withdrawal at home cages, and finally tested on a sensory preconditioning (SPC) paradigm. Two separate groups of rats performed either sucrose or cocaine, SA for 14 days. Following withdrawal, approximately half the animals in each SA group were assigned to a Treatment group and injected (i.p.) with either Vehicle or the D_3_R antagonist VK4-116 (15 mg/kg) 30 min before each session of the SPC paradigm. This created four separate SA × Treatment groups: Suc_Veh, Coc_Veh, Suc_D3a, Coc_D3a (final group numbers indicated). *Design of the sensory preconditioning paradigm:* (inset) In preconditioning (stage 1), rats were exposed to the sequential relationship between pairs of auditory stimuli A- > B and C- > D on separate trials. This was followed by conditioning (stage 2), where cue B was reinforced (3× sucrose pellets delivered during cue B presentation) whereas cue D was not reinforced. Finally, rats received two probe tests where cues A and C (test 1) or cues B and D (test 2; fixed test order) in extinction i.e. non-reinforced. The SPC effect: Greater anticipatory responding to A than C during the probe test indicates integration of learning across stages 1 and 2 i.e. A- > B->Pellet, C- > D->No-Outcome. The conditioning effect: greater anticipatory responding to cue B than D during the probe test indicates successful discriminative Pavlovian conditioning during stage 2. This extinction probe removes the pellet consumption responses that co-occur with anticipatory responding to the reinforced cue B during stage 2 conditioning. *Self-Administration training:* Rats were first trained on self-administration (SA) to press the active lever for either 10% sucrose liquid or cocaine (0.75/mg/kg/infusion). Rats in both the **B** sucrose and **C** cocaine groups successfully increased responding on the active lever, but not the inactive lever over 14 days of training. Overall, SA was acquired faster in the Sucrose group. Lever responses and reinforcer infusions are presented as a percentage of the maximum number of available infusions per session (max infusions was 60 for most animals; see “Methods” for details). SA plotted separately for males and females in Supplementary Fig. 1. Error bars depict mean ± SEM.

The ability to make associative inferences, an aspect of insight-based behavior, was tested using the SPC procedure. The SPC procedure consisted of three stages, described below (Fig. 1A). The stimuli were four distinct 10 s auditory cues, A and C (clicker and white noise, counterbalanced), B and D (tone and siren, counterbalanced). During conditioning, reinforcement entailed the delivery of 3 sucrose pellets within the 10 s presentation of stimulus B, such that a pellet was delivered at 3, 6.5, and 10 s. Immediately prior to preconditioning, rats were first shaped to receive food pellets from the magazine in a single session with 16 reinforcers (two sucrose pellets) delivered on a variable time schedule (*M* = 120 s ± 60 s).

1. Preconditioning: Rats received two days of preconditioning where two distinct S_1_-S_2_ stimulus pairs were presented: A- > B and C- > D. A stimulus pair consisted of a 10 s auditory stimulus (clicker or white noise; A or C, counterbalanced) immediately followed by a second 10 s auditory stimulus (tone or siren; B or D, counterbalanced). Each session consisted of a 6-trial block with one of the stimulus pairs (e.g. A- > B), followed by a second block of the other stimulus pair (e.g. C- > D). Block order was reversed on the second session, and fully counterbalanced across rats.
2. Conditioning: After 2 days of preconditioning, rats received 6 days of Pavlovian conditioning. Each session involved six reinforced trials of cue B (3 sucrose pellets delivered during cue presentation at 3, 6.5, and 9 s), and six non-reinforced trials of cue D (no pellets delivered), presented in pseudo-random trial order.
3. Probe test: After conditioning, rats received 2 days of non-reinforced probe tests i.e. in extinction. On the first day, the probe test included a total of 6 trials of cue A and 6 trials of cue C, presented in alternating blocks of three trials of each stimulus (order counterbalanced across rats). On the second day, the probe test included a total of 6 trials of cue B (non-reinforced) and 6 trials of cue D, presented in pseudo-random order. The order of the probe test sessions was fixed for all rats (testing A/C on day 1, and B/D on day 2).

The SPC effect is defined in the present task as greater responding to A than C during the Probe test, reflecting greater expectation of pellet rewards after A than C. Greater responding to A than C reflects the integration of learning about the cues across Preconditioning (stage 1) and Conditioning (stage 2). i.e. if A- > B and B->Pellet, then A- > B->Pellet, whereas if C- > D and D->Nothing, then C- > D->Nothing, therefore the expectation of reward should be greater during A than C. Importantly, A never directly predicts reward delivery, and is only associated with reward indirectly via its predictive relationship to B.

The study aimed to include approximately *n* = 16 rats per group to achieve significant statistical power to detect the predicted treatment effect (based on pilot data and published effect sizes using similar parameters) [8, 10]. This was achieved after training 5 cohorts. Analysis of the excluded animals did not reveal any systematic bias towards group membership or sex. Rats were excluded if they developed health issues during the experiment (Female *n* = 4, Male *n* = 5) if their catheter lost patency during SA training for cocaine and/or if they received fewer than 140 total infusions (cocaine or sucrose) over the 14 days of SA training (Female *n* = 7, male *n* = 4). Final group numbers were: Suc_Veh *N* = 18 (*n* = 7 Female, *n* = 11 Male), Suc_D3a *N* = 15 (*n* = 6 Female, *n* = 9 Male), Coc_Veh *N* = 14 (*n* = 6 Female, *n* = 8 Male), Coc_D3a *N* = 17 (*n* = 10 Female, *n* = 7 Male). Data were quantified and analyzed using standard procedures, similar to prior reports; see Supplemental Materials and Methods Online for full details.

## Results

### Self-administration

Rats successfully acquired SA behavior in both the sucrose and cocaine groups (Fig. 1B, C). Overall, responding on the active lever significantly increased over sessions, but not on the inactive lever (significant main effect of Session, $F\left(13,106.46\right)=9.18$, $p < .001$; Lever, $F\left(1,60.07\right)=98.61$, $p < .001$; and Lever × Session interaction, $F\left(13,112.30\right)=13.11$, $p < .001$; positive linear trend for the Active Lever, $t\left(264.63\right)=9.77$, $p < .001$; linear trend for the Inactive Lever, $t\left(264.63\right)=0.18$, $p=.854$). Acquisition of sucrose SA responding was faster than cocaine SA (main effect of SA, $F\left(1,60.08\right)=54.39$, $p < .001$; SA × Lever interaction, $F\left(1,60.07\right)=20.98$, $p < .001$; SA × Lever × Session interaction, $F\left(13,112.30\right)=3.17$, $p < .001$). Active lever responding increased significantly more quickly for the sucrose than the cocaine group (linear trend, Sucrose: Active Lever, $t\left(263.89\right)=9.20$, $p < .001$; Cocaine: Active Lever, $t\left(265.33\right)=4.66$, $p < .001$; significant SA × Session linear trend interaction on Active Lever, $t\left(264.63\right)=9.77$, $p < .001$); inactive lever responding did not differ (linear trend, Sucrose: Inactive Lever, $t\left(263.89\right)=0.66$, $p=.513$; Cocaine: Inactive Lever, $t\left(265.33\right)=-0.39$, $p=.699$; non-significant SA × Session linear trend interaction on Inactive Lever, $t\left(264.63\right)=0.18$, $p=.854$). Males acquired responding on the active lever faster than females in the Sucrose SA group, but no significant sex differences were evident in the Cocaine SA group (see Supplemental Analysis 1 for full analysis of sex differences). Importantly, SA acquisition was similar for animals assigned to the Vehicle and D3a treatment in the next stage of the experiment (analysis including Treatment as a factor; main effects and interactions with Treatment, all *p* > 0.118).

#### Stage 1 - Preconditioning

**Fig. 2:**
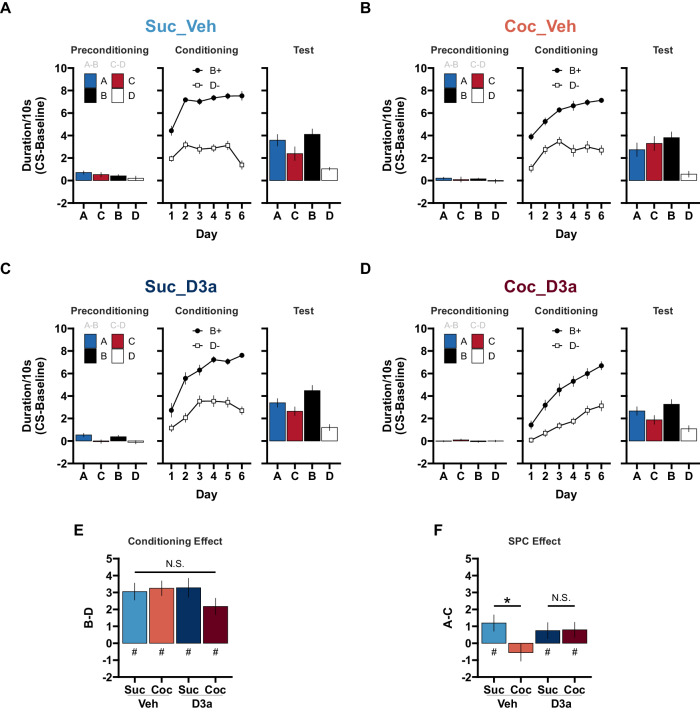
A history of cocaine self-administration disrupts sensory preconditioning (SPC) in rats treated with vehicle prior training sessions. The D_3_R antagonist VK4-116 effectively treats sensory preconditioning deficits in rats with a history of cocaine self-administration. Following SA training and 4 weeks of withdrawal, rats were trained on the SPC task. Prior to each session, rats were treated with either Vehicle or the D_3_R antagonist VK4-116 (15 mg/kg; i.p.). **A** Suc_Veh: Sucrose SA rats given Vehicle. **B** Coc_Veh: Cocaine SA rats given Vehicle. **C** Suc_D3a: Sucrose SA rats given VK4-116. **D** Coc_D3a: Cocaine SA rats given VK4-116. Stage 1 - Preconditioning (A- > B, C- > D), Stage 2 - Conditioning (B- > Pellets, D- > No-outcome), and Probe test (A, C, B, D: in extinction) behavior are presented for each group (left, middle, right). Discriminative responding to the cues is depicted as the duration of time spent in the food cup during the CS, above the pre-CS baseline (mean ± SEM). **E** The Conditioning effect: The difference in responding to cues B and D during the probe test provides an index of the conditioning effect such that scores above 0 reflect successful conditioning. **#** = Significant Conditioning effects: Responding to B was greater than D in vehicle (main effect of Cue, Vehicle: Cue B > D, $t\left(56\right)=8.50$, $p < .001$) and D3a treated groups (D3a: Cue B > D, $t\left(55.90\right)=7.43$, $p < .001$). **N**.**S**. = Magnitude of Conditioning effects were not significantly different: Magnitude of Cue (B > D) differences did not interact with SA experience in Vehicle (Vehicle: SA × Cue $t\left(56\right)=0.66$, $p=.512$) or D3a treated groups (D3a: SA × Cue, $t\left(55.90\right)=-1.46$, $p=.149$). **F** The SPC effect: The difference in responding to cues A and C during the probe test provides an index of the SPC effect such that scores above 0 reflect successful SPC. Sex and individual differences in the conditioning and SPC effect are depicted in Supplementary Fig. 2. Difference scores were calculated as the difference in discriminative responding to each cue from the Probe test in A–D (mean ± SEM). **#** = The SPC effect (A > C) was significant in Suc_Veh (Suc_Veh: A > C, $t\left(56\right)=2.58$, $p=.013$) but not Coc_Veh rats (Coc_Veh: A vs C, $t\left(56\right)=-1.05$, $p=.297$), reflecting a significant SA × Cue interaction for S1 stimuli (Veh: SA × Cue, $t\left(56\right)=-2.51$, $p=.015$). This reflected a significant SPC effect across both Suc_D3a and Coc_D3a groups (D3a: SA × Cue, A > C, $t\left(55.90\right)=2.02$, $p=.048$). **N**.**S**. = The SPC effect was of similar magnitude in the Suc_D3a and Coc_D3a treated groups (D3a: SA × Cue, $t\left(55.90\right)=0.26$, $p=.797$). Overall, the depicted pattern of differences in E and F are supported by significant SA × Treatment × Cue × Stimulus interaction ($F\left(1,112\right)=5.90$, $p=.017$), and this interaction was decomposed using separate ANOVAs for each Vehicle and D3a Treatment conditions. Alternatively, separate Treatment × SA × Cue analyses of the stimuli in E and F were also consistent with the reported pattern of significance. The Conditioning effect (**E**) did not differ between groups (main effect of Cue: B > D $F\left(1,56\right)=124.61$, $p < .001$; no main or interaction effects between SA, Treatment, and Cue, *p* > 0.130). The SPC effect was also consistent with the reported pattern (SA × Treatment × Cue interaction approached significance, $F\left(1,56\right)=3.76$, $p=.058$). See Supplementary Analysis 4 for additional statistical support and considerations.

Overall, responding during exposure to the non-reinforced cue pairs in preconditioning was low (Fig. 2). Sucrose animals spent more time in the port than Cocaine SA animals (main effect of SA: Suc > Coc, $F\left(1,56\right)=6.69$, $p=.012$; no main effect of Treatment: Veh vs D3a, $F\left(1,56\right)=2.60$, $p=.113$; no SA × Treatment interaction, $F\left(1,56\right)=0.65$, $p=.422$). Responding was also higher to the first stimulus in each pair (main effect of Stimulus: S1 > S2, $F\left(1,56\right)=11.09$, $p=.002$), and slightly higher to the AB than the CD cues (main effect of Cue: AB > CD, $F\left(1,56\right)=4.19$, $p=.045$; but no Cue × Stimulus interaction, $F\left(1,56\right)=0.06$, $p=.811$). Importantly, there were no significant interactions between SA, Treatment, and Stimulus order or Cue pairs (all *p* > 0.060).

A full analysis of any sex differences in SPC is reported in the supplementary analyses; however, any observed significant sex differences were transient, and were not observed by the end of conditioning in stage 2, or during the critical probe tests.

#### Stage 2 - Conditioning

All four groups successfully increased responding to cue B more than cue D by the end of conditioning (main effect of Cue, $F\left(1,56\right)=726.65$, $p < .001$; Session, $F\left(5,560\right)=79.20$, $p < .001$; and significant Cue × Session, $F\left(5,560\right)=13.42$, $p < .001$). However, it is important to note that pellets were delivered during presentations of cue B, and responding in this stage reflects both anticipation and consumption of the pellets. Responding to cues B and D during the probe test (i.e. in extinction) can provide a test of any differences in anticipatory responding without this confound.

There was greater cue discrimination (B > D) in the sucrose than the cocaine SA groups (SA × Cue interaction, $F\left(1,56\right)=6.35$, $p=.015$; significant cue discrimination in both SA conditions, Sucrose: B > D, $t\left(56\right)=21.08$, $p < .001$; Cocaine: B > D, $t\left(56\right)=17.09$, $p < .001$), however there was also a greater linear increase in responding to both cues in the cocaine than sucrose groups (SA × Session interaction, $F\left(5,560\right)=5.39$, $p < .001$; significant positive linear trend in both SA conditions: Sucrose, $t\left(560\right)=9.60$, $p < .001$; Cocaine, $t\left(560\right)=14.92$, $p < .001$; Overall responding main effect of SA: Suc > Coc, $F\left(1,56\right)=14.42$, $p < .001$).

When looking at the effects of Treatment groups, there was greater cue discrimination (B > D) in the Vehicle than the D3a groups (Treatment × Cue interaction, $F\left(1,56\right)=8.21$, $p=.006$; significant cue discrimination in both Treatment conditions, Vehicle: B > D, $t\left(56\right)=21.04$, $p < .001$; D3a: B > D, $t\left(56\right)=17.07$, $p < .001$), however there was also greater linear increase in responding to all cues in the D3a than vehicle Treatment groups (Treatment × Session interaction, $F\left(5,560\right)=11.27$, $p < .001$; significant positive linear trend in both SA conditions, Vehicle: linear trend, $t\left(560\right)=7.19$, $p < .001$; D3a: linear trend, $t\left(560\right)=17.41$, $p < .001$; and an overall main effect of Treatment: Veh > D3a, $F\left(1,56\right)=12.40$, $p=.001$).

Overall, the effects of SA and Treatment did not interact significantly (SA × Treatment and higher order interactions with Cue and Session, *p* > 0.054).

##### Final session

Focusing on the final day of conditioning (Session 6), all groups responded significantly more to cue B than D (main effect of Cue, $F\left(1,56\right)=420.36$, $p < .001$). Between groups, there were no differences in responding to cue B, however responding to cue D was higher in the Cocaine than Sucrose SA groups (SA × Cue interaction, $F\left(1,56\right)=11.15$, $p=.002$; Cue B: Suc vs Coc, $t\left(102.22\right)=-1.47$, $p=.144$; Cue D: Suc < Coc, $t\left(102.22\right)=2.45$, $p=.016$), and greater in the D3a than the Vehicle Treatment groups (Treatment × Cue interaction, $F\left(1,56\right)=6.45$, $p=.014$; Cue B: Veh vs D3a, $t\left(102.22\right)=-0.37$, $p=.714$; Cue D: Veh < D3a, $t\left(102.22\right)=2.62$, $p=.010$). All remaining main effects and interactions between SA, Treatment, Cue, and Sex failed to reach significance (*p* > 0.100).

#### Stage 3 - Probe test

During the probe test (Fig. 2A–D), all groups demonstrated successful stage 2 conditioning (i.e., the conditioning effect, B > D; Fig. 2E). In contrast, all groups except for the untreated cocaine group (Coc_Veh), responded more to cue A than C (i.e. the SPC effect, A > C; Fig. 2F). This pattern of group differences was supported by a significant SA × Treatment × Cue × Stimulus interaction ($F\left(1,112\right)=5.90$, $p=.017$; a main effect of Cue pair, AB > CD, $F\left(1,112\right)=89.16$, $p < .001$; and a Cue × Stimulus interaction, $F\left(1,112\right)=41.78$, $p < .001$; all remaining effects, including sex differences did not reach significance, *p* > 0.098; Sex and individual subject data points corresponding to Fig. 2E, F are presented in Supplementary Fig. 2), and explored with separate follow-up analyses within each Treatment condition (additional analysis within each SA condition presented in Supplementary Analysis 4).

##### Vehicle Treatment

In the Vehicle-treated groups, sucrose SA rats showed a significant SPC effect that was abolished in cocaine SA rats. This was supported by a significant SA × Cue × Stimulus interaction ($F\left(1,56\right)=5.01$, $p=.029$), such that the SPC effect (A > C) was significant in Suc_Veh (Suc_Veh: A > C, $t\left(56\right)=2.58$, $p=.013$) but not Coc_Veh rats (Coc_Veh: A vs C, $t\left(56\right)=-1.05$, $p=.297$; reflecting a significant SA × Cue for S1 stimuli, S1: SA × Cue $t\left(56\right)=-2.51$, $p=.015$). Importantly, both groups showed significant evidence of successful discriminative responding to the previously rewarded cue B compared to the non-reinforced cue D (Suc_Veh: B > D, $t\left(56\right)=5.88$, $p < .001$; Coc_Veh: B > D, $t\left(56\right)=6.15$, $p < .001$), and this effect was of similar magnitude in both groups (no SA × Cue interaction for S2 stimuli, S2: SA × Cue $t\left(56\right)=0.66$, $p=.512$). This finding successfully replicates our earlier report that a history of cocaine SA disrupts SPC in rats, and it further extends this by suggesting that there are no significant sex differences in this effect (Supplementary Fig. 2).

##### D3a treatment

In D3a treated groups, the SPC effect was significant and did not differ between sucrose and cocaine SA rats. This was supported by a significant Cue × Stimulus interaction ($F\left(1,28\right)=15.25$, $p=.001$; and significant main effect of Cue pair, AB > CD, $F\left(1,28\right)=42.89$, $p < .001$) that did not differ between Suc_D3a and Coc_D3a groups (no Treatment × Cue × Stimulus interaction, $F\left(1,28\right)=1.55$, $p=.224$). This reflected a significant SPC effect across both Suc_D3a and Coc_D3a groups (A > C, $t\left(55.90\right)=2.02$, $p=.048$), as well as a significant, albeit larger, effect of discriminative responding (B > D, $t\left(55.90\right)=7.43$, $p < .001$). Notably, both of these effects were of similar magnitude in both groups (no SA × Cue interaction for S1 or S2 stimuli; SPC effect, S1: SA × Cue, $t\left(55.90\right)=0.26$, $p=.797$; conditioning effect, S2: SA × Cue, $t\left(55.90\right)=-1.46$, $p=.149$). Therefore, treating cocaine-experienced rats with the D3-receptor antagonist VK4-116 successfully recovered deficits in SPC (see also Supplementary Analysis 4).

#### Group differences in SPC task interpretation

Although no cues were used in our self-administration sessions, it is nevertheless plausible that different histories of cocaine or sucrose SA (i.e. Suc_Veh and Coc_Veh) could change the nature of how the cues are represented, integrated, or generalized across the stages of the SPC task, i.e. how the task is “solved”. In control animals, the SPC effect can be supported by a number of mechanisms that are likely to be a parameter dependent [41, 42]. In the present version of the task, the predominant mechanism in controls is likely to be associative inference during the probe test [9, 43] i.e. at test, responding to cue A is driven by recall of the A- > B (stage 1) and B->Pellet (stage 2) associations that are integrated to infer that A- > B->Pellet. This is suggested by several lines of evidence, including the OFC-dependence of responding in the probe test [8, 44], as well as the failure of the preconditioned cue to support conditioned reinforcement [43], both of which are contrary to other mechanisms, especially mediated learning, in which value would accrue directly to the preconditioned cue [41]. This solution to SPC is disrupted by a history of cocaine use, both here in the Coc_Veh group as well as in our prior study [10]. If VK4-116 (D3a) is successfully “treating” the effect of Cocaine SA, then the Coc_D3a group should also have a task solution that is more similar to the Suc_Veh than the Coc_Veh groups. We tested the similarity of the group solutions using two complementary approaches: (1) testing specific predictions about the A~B and C~D relationships in each group, and (2) comparing the similarity of the overall behavioral task solution between groups.

To test this, we first examined probe test behavior to see if responding to cues A and C was related to the level of conditioning to cues B and D, i.e. is there an S1- > S2 relationship? Specifically, in the Suc_Veh group we expected a strong positive correlation between learning about reinforced cue B and responding to cue A and no correlation between non-reinforced cue D and cue C during the probe test (reflecting low levels of magazine responding to cue D that are likely driven by factors other than reward expectation). In contrast, in the Coc_Veh group we expected that responding would not be correlated between cues pairs A-B and C-D. Importantly, we predicted that the D3a treatment in the Coc_D3a group would recover the strong positive correlation between responding to cues A and B, but not C and D. The pattern of group differences in slopes between cues A~B and C~D was compared by fitting a linear model predicting responding to the first stimulus in each pair (S1: A/C) with responding to the relevant second cue in each pair (S2: B/D), as well as categorical factors of Cue pair (AB/CD), Treatment, and SA.

**Fig. 3:**
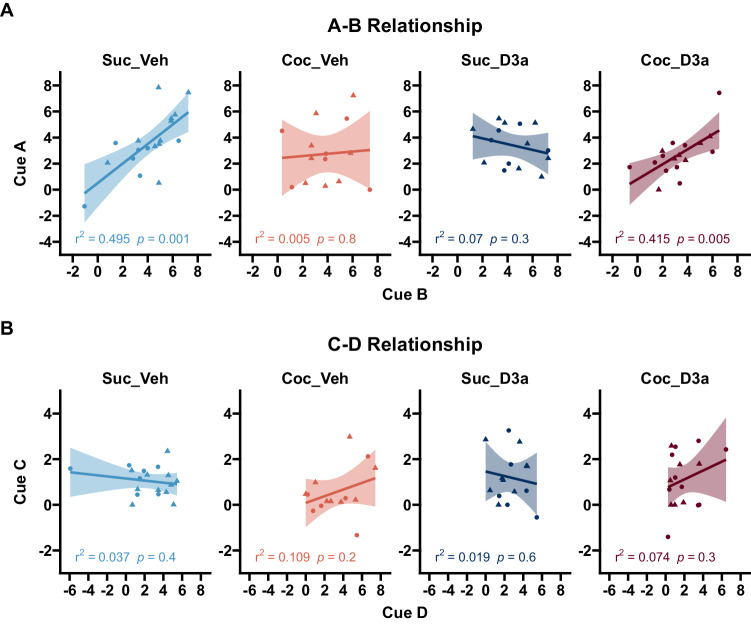
Testing whether probe test responding reflects the relationships between the cue pairs that were presented during stage 1 preconditioning and stage 2 conditioning (A- > B->Pellet, C- > D->No-outcome). **A** *A–B relationship:* Responding to cue A is driven by learning to B in both the control group (Suc_Veh) and treated cocaine group (Coc_D3a), but not in the untreated cocaine (Coc_Veh) and treated control groups (Suc_D3a). **B** *C-D relationship:* There was no relationship between responding to cue C and D. Data points indicate probe test responding for individual subjects for the first cue (y-axis) and second cue (x-axis) in each pair, and plotted separately for each group (panels from left to right). Responding was defined as the duration of time spent in the food cup during the CS, above the pre-CS baseline. Lines and error shading from linear model fit, and corresponding model *r*^2^ and *p*-values presented at the bottom of each plot. The sex of individual subjects is represented as: Female = Circle, Male = Triangle.

Consistent with our predictions (Fig. 3A), there was a significant positive correlation between A and B in the Suc_Veh and Coc_D3a groups but not in the Coc_Veh group or, unexpectedly, in the Suc_D3a group (Suc_Veh: A~B, $b=0.75$, 95% CI $\left[0.35,1.15\right]$, $t\left(16\right)=3.96$, $p=.001$; Coc_D3a: A~B, $b=0.57$, 95% CI $\left[0.20,0.95\right]$, $t\left(15\right)=3.26$, $p=.005$; Coc_Veh: A~B, $b=0.09$, 95% CI $\left[-0.66,0.83\right]$, $t\left(12\right)=0.25$, $p=.805$; Suc_D3a: A~B, $b=-0.22$, 95% CI $\left[-0.70,0.26\right]$, $t\left(13\right)=-0.99$, $p=.340$). These slope differences between SA and Treatment groups was supported by a significant SA × Treatment interaction (A~B: SA × Treatment, $F\left(1,56\right)=9.68$, $p=.003$), which reflected significant differences between slopes for Coc_Veh and Suc_Veh groups (A~B: Coc_Veh < Suc_Veh, $t\left(56\right)=-2.10$, $p=.040$), and Coc_D3a and Suc_D3a groups (A~B: Coc_D3a > Suc_D3a, $t\left(56\right)=2.30$, $p=.025$).

In contrast to the A~B correlations, there were no significant relationships between cue C and D in any group (Fig. 3B; Suc_Veh: C~D, $b=-0.81$, 95% CI $\left[-3.01,1.38\right]$, $t\left(16\right)=-0.79$, $p=.443$; Coc_Veh: C~D, $b=0.74$, 95% CI $\left[-0.59,2.08\right]$, $t\left(12\right)=1.21$, $p=.249$; Suc_D3a: C~D, $b=-0.19$, 95% CI $\left[-1.01,0.63\right]$, $t\left(13\right)=-0.50$, $p=.625$; Coc_D3a: C~D, $b=0.38$, 95% CI $\left[-0.36,1.11\right]$, $t\left(15\right)=1.09$, $p=.292$). The lack of significant group differences in the C-D relationship was consistent with a non-significant SA × Treatment interaction (C~D: SA × Treatment, $F\left(1,56\right)=0.68$, $p=.414$; all remaining main effects and interactions with cue D, *p* > 0.399). Finally, this overall pattern of group differences in slopes between cues A~B but not C~D was supported by a significant SA × Treatment × Cue × S2 interaction ($F\left(1,112\right)=4.06$, $p=.046$).

These results support our predictions that responding to cue A was uniquely related to learning about cue B in control rats (Suc_Veh), which was disrupted by a history of cocaine use (Coc_Veh) but successfully recovered by D3a treatment (Coc_D3a). Surprisingly, the D3a treatment disrupted the A~B relationship in sucrose-control rats (Suc_D3a) but not the SPC effect. This result is important because it indicates that the mitigation of the cocaine-related deficits is not due to an independent effect of VK4-116 to somehow improve normal performance in preconditioning. Indeed, it supports the idea that VK-116 is having an effect in the cocaine-trained group that reflects an interaction with changes caused by cocaine use.

#### Behavioral similarity

**Fig. 4:**
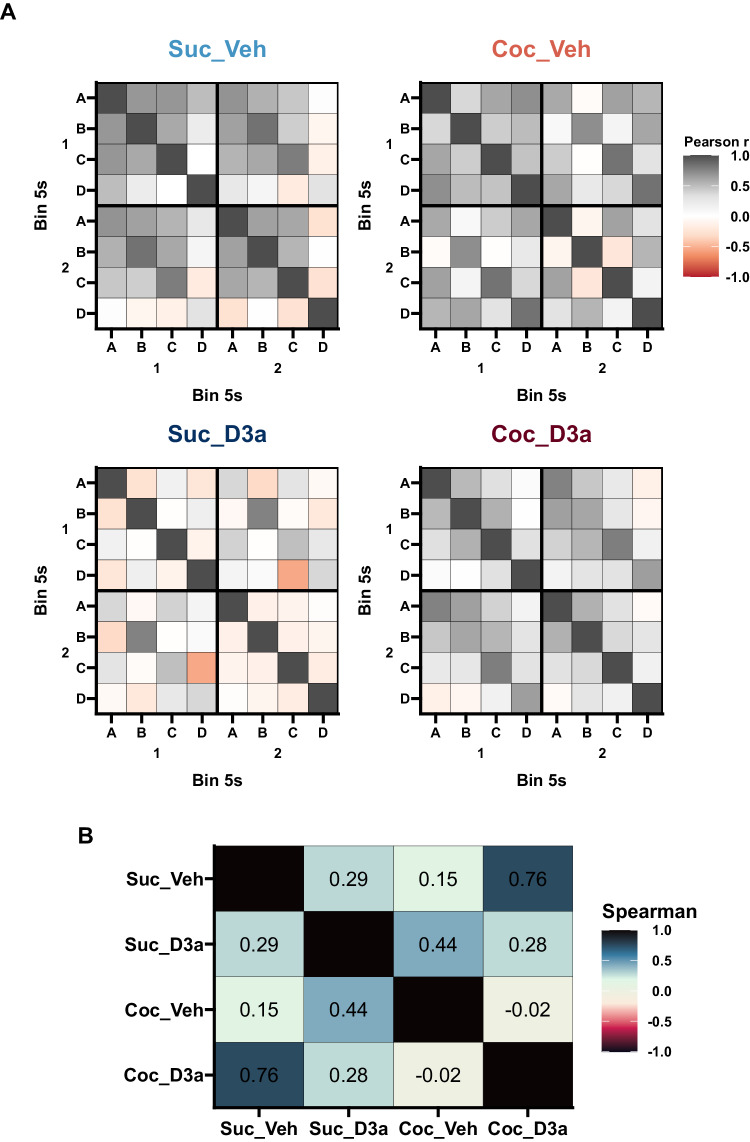
Behavioral similarity analysis of probe test responding. **A** Behavioral similarity matrices for Sucrose (left) or Cocaine (right) SA groups, and Vehicle (top) or D_3_R antagonist (bottom) treatment groups. For each group, the similarity of responding between and within cues was quantified by separating responses within each cue into 5 s time bins (Supplementary Fig. 3) and generating a cross-correlation matrix between all the resultant cue epochs. Color values are plotted to represent the correlation values (Pearson’s r), to provide a visual summary of the group specific pattern of response relationships. The behavioral similarity patterns were similar between the untreated control group (Suc_Veh) and the cocaine group treated with the D_3_R antagonist (Coc_D3a), but different to the untreated cocaine group (Coc_Veh) and the control group treated with the D_3_R antagonist (Suc_D3a). **B** *Behavioral similarity analysis:* A Spearman correlation was used to test the similarity between the group similarity matrices. Numbers (and corresponding color values) indicate the rank correlation (Spearman’s rho) between the lower diagonal of the cross-correlation matrices above. An analysis of just the between-cue similarities (i.e. not considering within-cue time bins) supported similar conclusions (Supplementary Fig. 4).

Next, we tested the similarity of the overall task solutions in each group using a behavioral similarity analysis, an approach based on representational similarity analysis [45]. How a task is solved is likely to be reflected in the pattern of multivariate relationships both between and within each cue. To capture the pattern of responding within each cue, the 10 s cue period was split into early and late epochs (i.e. 5 s bins), a standard approach [46–48] that also reflected the observed pattern of responding within-cue (Supplementary Figs. 3–4). A Pearson cross-correlation matrix was calculated across all 8 (cue [4] × time [2]) epochs to create a behavioral similarity matrix for each group (Fig. 4). These behavioral similarity matrices represent the multivariate patterns of probe test responding within each group, i.e. a group level index of the overall task solution. Finally, we tested whether groups used similar overall task solutions by comparing behavioral similarity matrices between pairs of groups using Spearman’s rank correlations. Evidence of significant similarity was found between Suc_Veh and Coc_D3a groups (Spearman’s rank correlation, Suc_Veh vs Coc_D3a, ${r}_{{{{{{\rm{s}}}}}}}=.76$, $S=894.00$, $p < .001$), but not between the other groups (Suc_Veh vs Coc_Veh, ${r}_{{{{{{\rm{s}}}}}}}=.15$, $S=3,088.00$, $p > .999$; Suc_Veh vs Suc_D3a, ${r}_{{{{{{\rm{s}}}}}}}=.29$, $S=2,594.00$, $p=.805$; Coc_Veh vs Coc_D3a, ${r}_{{{{{{\rm{s}}}}}}}=-.02$, $S=3,716.00$, $p > .999$; Coc_Veh vs Suc_D3a, ${r}_{{{{{{\rm{s}}}}}}}=.44$, $S=2,052.00$, $p=.123$; Coc_D3a vs Suc_D3a, ${r}_{{{{{{\rm{s}}}}}}}=.28$, $S=2,622.00$, $p=.871$; Holm-Sidak corrected). These findings provide further evidence that D3a treatment returned the performance of cocaine-experienced rats to what is normally observed, and that this effect was selective, representing an interaction with changes caused by cocaine use, and not simply an independent effect of VK4-116 to improve normal inference in the preconditioning task.

## Discussion

A history of cocaine use in humans and other animals is associated with behavioral changes linked an increase in mesolimbic D_3_R availability, and a lack of behavioral insight, i.e., an inability to use learned information to mentally simulate and make inferences about cause and effect. The present study tested whether the novel selective D_3_R-antagonist, VK4-116, could effectively treat impaired behavioral inferences in sensory preconditioning (SPC) in rats following withdrawal from cocaine self-administration.

We found that a history of cocaine self-administration caused a significant impairment in the ability of vehicle-treated rats to make inferences by integrating prior knowledge about the relationships between cues and outcomes in the SPC task. However, treating cocaine-experienced rats with the D_3_R-antagonist VK4-116 effectively restored inference and a pattern of behavior in the task strikingly similar to vehicle-treated sucrose-control rats. This was evident in two metrics of responding to cue A and C at test that reflect the inference that A- > B->Reward and C- > D->Nothing: (1) responding more to cue A than C, and (2) the strength of responding to cue A reflected the strength of responding to cue B. Surprisingly, VK4-116 also partially disrupted inference in sucrose-experienced control rats such that while responding to A was greater than C, it was unrelated to responding to cue B. Importantly, these cocaine and VK4-116 treatment effects were not simply due to differences in learning or responding to cues B and D, i.e. non-inference-based behavior. Overall, these findings suggest that both VK4-116 and cocaine history shifted how the SPC task was interpreted. These data provide evidence that D_3_R play a key role in the loss of behavioral insight and inference processes following cocaine use and suggest VK4-116 as a promising pharmacotherapy to selectively treat these symptoms in CUD.

### D_3_R function is related to impaired inference following cocaine use

The selective D_3_R-antagonist VK4-116 impaired inference in drug-naïve rats but effectively restored it in rats after prior cocaine exposure that has previously been shown to increase brain D_3_R availability [27]. This bidirectional effect of VK4-116 suggests that D_3_R plays a causal role in modulating inference and that increased midbrain D_3_R availability is a key factor in impaired behavioral inference following cocaine use. This account is consistent with the extant literature on the role of D_3_R in cocaine use and its behavioral sequalae (Newman et al.). In drug-naïve rodents, higher levels of midbrain D_3_R availability predicts deficits in probabilistic reversal learning tasks, as well as increased motivation and escalation of subsequent cocaine SA [27, 28]. Furthermore, pharmacological activation of D_3_R with the D_3_R-preferring agonist pramipexole, in drug-naïve rats, exacerbates probabilistic reversal learning deficits in a manner similar to cocaine use [28]. While there is evidence that individuals with high levels of D_3_R availability are more likely to use cocaine, subjects with cocaine experience exhibited increased D_3_R availability in the striatum and midbrain in humans and non-human primates [12–17]. In the present study, we found that pretreatment with a selective D3R antagonist (VK4-116) reversed the cocaine-induced deficit in inference in rodents, suggesting that higher D3R availability in the brain could be a risk factor in the development of cocaine use disorder.

In considering the significance and translatability of these findings, it is important to consider several aspects of the experiment. First, we did not assess effects of VK4-116 on drug use directly [36], nor did we relate its effects on SPC to drug-seeking. Given that the criteria for SUD generally reflect an inability to use non-drug outcomes to modulate behavior and given that many of these non-drug outcomes are probabilistic, delayed, or even anecdotal, often reflecting learning that has occurred in contexts other than the drug-seeking [49], we believe it is reasonable to speculate that deficits in inference in a controlled setting like SPC would predict difficulties in using such knowledge to control drug-seeking. While this has not been directly shown here or to the best of our knowledge elsewhere, this would be an important next step.

Second, we used a relatively short period of drug use and assessed SPC after a relatively long period of forced abstinence. The use of short rather than extended access, or other models of individual variability in SUD, was intentional and reflects the hypothesis that changes in brain function underlying effects on behavior like that shown here are a contributing factor, one of multiple hits, which lay the groundwork for progression to SUD. Testing after a period of abstinence was also intentional, since we are interested not in directly disrupting responding for drug, which can be accomplished by a variety of interventions in rats and humans [50–54], but rather in developing ways to address longer term more difficult to address phenomena such as relapse and reinstatement [55].

Finally, we recognize that SPC consists of three separate learning phases, and we pre-treated with VK4-116 prior to learning in each phase. This reflected the fact that changes in brain function due to prior drug use potentially impacts learning and changes in associative representations occurring in all three phases, and the growing realization that brain areas involved in inference may play critical roles in each of the three phases [41, 56]. For example, the OFC has long been associated with performance in the final probe phase of tasks such as SPC and devaluation, where its function to allow inference of outcomes “on the fly” via the use of model-based representations was thought to be important. However, it is now appreciated that the orbitofrontal cortex also plays a role in the formation of those representations during initial learning [57]. For example, we have shown that inactivation of the OFC in the initial phase of learning in preconditioning and devaluation disrupts later use of the latent information in the probe test, even if OFC is back online. Clearly it may be useful in the future to determine if selective D_3_-receptor antagonists, such as VK4-116, are critical in one particular phase, or even to dissociate its administration from the entire task in time as the therapeutic effects might be treating underlying circuitry imbalances related to dopaminergic dysregulation (discussed below). However, while answers to these questions are important, they are not necessary to appreciate the potential therapeutic significance of the current results.

### Relationship between D_3_R and orbitofrontal function in CUD

There appears to be a strong connection between midbrain D_3_R and OFC function. D_3_Rs are inhibitory G-protein coupled receptors. Activation of D3Rs in dopamine neurons in substantia nigra and ventral tegmental area inhibits dopamine release in striatum and OFC [21–25]. Consistent with this relationship, imaging studies also indicate that OFC hypoactivity is a consistent feature in abstinent cocaine users, and is correlated with increased D_3_R availability in substantia nigra [20, 58, 59]. In non-clinical populations, midbrain D_3_R availability is also correlated with both resting state OFC activity, and the functional connectivity between OFC subregions and multiple key large-scale neural systems such as salience executive control networks, basal ganglia/limbic network, and the default mode network [26]. Thus far, it is unknown which neuronal cell type displays D_3_R up-regulation in subjects with CUD. If it occurs mainly in midbrain DA neurons, blockade of abnormal D_3_Rs would disinhibit (increase) dopamine neuron activity, which may subsequently increase OFC neuronal activity and improve functional connectivity between the OFC and other networks. This may in part underlie the therapeutic effects of D_3_R antagonism on cocaine-induced impairment of behavioral inference.

Recent evidence from cocaine self-administration and forced abstinence studies in rodents and non-human primates also supports a causal link between cocaine use, increased brain D_3_R availability, OFC hypofunction, and behavioral deficits in tasks like probabilistic reversal learning [27–29]. Indeed, there is significant overlap between the behavioral deficits following cocaine use and OFC dysfunction, including in tasks as diverse as reversal learning, SPC, Pavlovian over-expectation, and outcome devaluation [60].

We have previously shown that cocaine experience disrupts OFC function and impairs insight-based behavior in rats, and optogenetic activation of pyramidal neurons in OFC can successfully restore insight-based behavior [7]. These findings parallel the present study and suggest that increased midbrain D_3_R availability and OFC hypofunction are correlated and underlie deficits in insight-based behavior following cocaine use. Treatment with the selective D_3_R-antagonist VK4-116 may well have its effect in the cocaine-experienced rats here through restoring the normal balance in dopamine effects on OFC function altered by drug experience. Consistent with the possibility of such long-term adaptations, VK4-116 significantly reduces self-administration of oxycodone in rats when co-administered over 5 days, and this effect persists for a couple of days in the absence of VK4-116 [34].

### Summary

Here we show the first evidence for the efficacy of VK4-116, a novel and highly selective D_3_R antagonist, in the treatment of deficits in inference-based behavior caused by cocaine use. These findings support established evidence in rodents that D_3_R antagonists significantly reduce drug-taking and relapse in rodent models of cocaine, nicotine, and oxycodone self-administration [30, 31, 34]. In contrast to antagonists that target D_2_Rs, VK4-116 does not appear to significantly disrupt overall activity levels or cause anhedonia [34]. Therefore, selective D_3_R antagonists such as VK4-116 are a promising therapeutic target for CUD and other SUDs [27, 38].

### Supplementary information

Supplemental Material
