# Supplementary material for: The selective D3Receptor antagonist VK4-116 reverses loss of insight caused by self-administration of cocaine in rats
Source: Neuropsychopharmacology. 2024 Apr 6;49(10):1590–9. doi: 10.1038/s41386-024-01858-7 (PMC11319511; doi:10.1038/s41386-024-01858-7)
Supplement: Supplementary file 1 — Supplemental Material [file 41386_2024_1858_MOESM1_ESM.docx]

# Supplementary Materials and Methods

## Subjects

Subjects were 45 female and 45 male (N = 90) Long-Evans rats (250-300g, ~3 months old) (Charles-River Laboratories, Wilmington, MA, USA) housed individually upon arrival, maintained on a 12hr light:dark cycle, and tested during the light phase. All testing procedures were approved by the NIDA-IRP Animal Care and Use Committee. Rats were given at least 7 days to acclimate to the animal facility with *ad-libitum* access to food and water prior to any experimental procedures. Rats were food restricted 3 days prior to behavioral testing and pre-exposed to the relevant food reinforcer (10% w/v sucrose liquid and Banana flavored sucrose pellets (Bio-Serv, Frenchtown, NJ), self-administration and sensory preconditioning training respectively), but returned to *ad-libitum* food access during the forced cocaine withdrawal period. During food restriction, rats were fed at the end of each testing day (approximately 10 or 15g per female and male rat, respectively), and weights were maintained above 85% of their baseline body weight. The experiment was conducted in 5 separate cohorts run sequentially: Cohort 1 (n = 6 Female, n = 6 Male), Cohort 2 (n = 6 Female, n = 6 Male), Cohort 3 (n = 6 Female, 6 Male), Cohort 4 (n = 12 Female, n = 12 Male), Cohort 5 (n = 15 Female, 15 Male). Experiments were performed at the National Institute on Drug Abuse, Intramural Research Program (NIDA-IRP), in accordance with the US National Institutes of Health (NIH) guidelines and were approved by the NIDA-IRP Animal Care and Use Committee (ACUC).

## Apparatus

Self-administration was conducted in standard modular rodent behavior boxes (Cohorts 1-3: Med Associates Inc., Georgia, VT, USA; Cohorts 4-5: Coulbourn Instruments, Allentown, PA, USA) inside sound attenuating chambers. Self-administration boxes were equipped with a retractable lever and a non-retractable lever on the right and left side of the front wall (designated as the active and inactive lever, respectively), and a house light centered at the top of the back wall. For cocaine self-administration, the back mounted catheter harness was connected to silastic tubing encased in a metal spring that was mounted to a swivel arm above the operant box and connected to an infusion pump mounted in the sound attenuating chamber (Instech Laboratories, PA, USA). For sucrose self-administration the boxes were equipped with a recessed magazine located between the two levers that provided access to 0.04 mL of sucrose liquid (10% w/v) via a retractable dipper cup.

Sensory preconditioning training was conducted using the same type of standard rodent behavior boxes (Coulbourn Instruments, Allentown, PA, USA) in sound attenuating chambers but located in a separate room. These boxes were equipped with a recessed food magazine in the center of the front wall, connected to a pellet dispenser mounted on the outside of the box that could deliver 45 mg banana flavored sucrose pellets (F0024, Bio-Serv, NJ, USA) into a food cup at the base of the magazine. Auditory stimuli could be delivered via a clicker mounted to the top-right of the front wall, a speaker on the opposite (top-left) of the back wall connected to a white-noise generator, and a speaker mounted on the top-right of the back wall connected to a tone generator. The auditory stimuli were a clicker (2 Hz), white noise (~70 dB), a steady tone (1500 Hz, ~75 dB), or a siren (oscillating at 5 Hz between 1000 Hz and 2000 Hz tones, ~75 dB).

## Surgical procedures

Prior to behavioral testing, chronic jugular catheters were implanted under aseptic conditions [1] . Rats were anaesthetized with either ketamine (100 mg/kg, i.p., Sigma) and xylazine (10 mg/kg, i.p., Sigma) (Cohorts 1-3), or isoflurane gas (induction at 4% and maintained at 1-2%, 2L/min O_2_) (Cohorts 4-5). A 22-gauge catheter (part# C30PU-RJV1412, Instech Laboratories, PA, USA) was inserted into the jugular vein and passed through an incision on the rat’s back to connect to a vascular access harness (part# VAH95AB14, Instech Laboratories, PA, USA). Some rats assigned to the sucrose self-administration condition received sham surgery in which the jugular vein was exposed but no catheter was implanted. Rats recovered for at least 7 days prior to self-administration training. Catheters were flushed daily with 0. 1 ml of mixture solution of gentamycin (1 mg/ml) and heparinized (1000 unit/ml) saline to maintain patency. Prior to self-administration training, catheter patency was assessed by observing loss of muscle tone following a brief methohexital (brevital) challenge (0.1 ml of 10 mg/ml brevital, i.v. via the catheter). Rats with patent catheters were then assigned to the cocaine self-administration groups, and the remaining rats with patent/non-patent catheters or sham surgeries were assigned to the sucrose self-administration groups.

## Drugs

Cocaine-HCL (NIDA IRP, Baltimore, MD, USA) was dissolved in saline, and administered through i.v. catheters at a concentration of 0.75 mg/kg/infusion (0.1 ml per infusion). To account for the range of rat weights during self-administration training, rats were weighed each day and assigned to a 20g weight interval (e.g. 300 – 320g), and received a cocaine-HCL concentration corresponding to the average of the weight band (e.g. 310g). Weights remained relatively stable, and the rats did not need to change cocaine-HCL concentrations during self-administration training.

VK4-116 [(±)-N-(4-(4-(3-chloro-5-ethyl-2-methoxyphenyl)piperazin- 1-yl)-3-hydroxybutyl)-1H-indole-2-carboxamide] was synthesized using the published method [2], and dissolved in vehicle to be administered at a dose of 15 mg/kg. This dose was selected as the optimal dose based on previous work showing that it successfully disrupts oxycodone self-administration while leaving oral sucrose self-administration intact in Long-Evans rats [3].Vehicle was 25% (w/v) 2-hydroxypropyl-β-cyclodextrin (vendor#: 332607; Sigma-Aldrich St. Louis, MO, USA) dissolved in distilled water. VK4-116 and vehicle injections were administered i.p. at a volume of 1 ml/kg. All drugs were prepared fresh and kept for up to five days refrigerated.

## Self-administration (SA)

One day prior to SA, rats assigned to the sucrose condition were given one pretraining session with two phases to familiarize them with the sucrose dipper cups. During phase one (1 hr) the sucrose dipper was presented non-contingently for 40s approximately every 80s (variable inter-reinforcer interval schedule). During phase two (1 hr) the active lever was inserted into the chamber for a maximum of 30 trials in which an active lever press resulted in lever retraction, 40s sucrose dipper presentation, and an 80s inter-trial interval.

Following this, rats were trained to self-administer intravenous cocaine-HCl (0.75 mg/kg/infusion, 4s per infusion) or oral sucrose (0.05 ml, 10%, w/v) under a fixed ratio 1 schedule (FR1), in a 3-hr session/day for 14 consecutive days. Each session consisted of trials where the active lever was inserted, signaled the availability of a reinforcer. A single response on the active lever (FR1) resulted in the retraction of the active lever, and either a 4s presentation of the sucrose dipper (0.05 mL sucrose 10% w/v) or a 4s drug infusion (0.75 mg/kg). This was followed by a 40s timeout period (inter-trial interval) where the active lever was kept retracted, and no reinforcers were available. A non-reinforced lever was always extended and present throughout the entire session, and responding on this lever was recorded but had no programmed consequences. Within each hour, rats could earn a maximum of 20 reinforcers and had at least 15 mins timeout with no reinforcers i.e. up to 45 min to complete 20 trials. This procedure ensured that cocaine rats received a maximum of 20 infusions per hour and 60 infusions per session/day to prevent overdose. Due to experimenter error, one cohort of rats (Cohort 1) in the sucrose SA condition could earn up to 100 reinforcers per hour.

## Withdrawal period

Following the completion of SA training, all rats were returned to *ad libitum* food access in their home cages with no experimental training for least 4 weeks of the withdrawal. Catheters and harnesses were removed at the start of the withdrawal period by cleaning and cutting the tubing such that it was no longer exposed. All rats received prophylactic treatment of enrofloxacin (0.454%, 1 ml, s.c.) for three days following catheter removal.

## Treatment

Rats in the sucrose (Suc) and cocaine (Coc) SA conditions were pseudo-randomly allocated to either a vehicle (Veh) or D_3_R antagonist (D3a) treatment condition to create a total of four groups: Suc_Veh, Coc_Veh, Suc_D3a, and Coc_D3a. Pseudo-random allocation was used to roughly match the rates of SA behavior in each group, and equate the number of males and females. Treatment involved an i.p. injection of VK4-116 (15 mg/kg; D3a) or vehicle (25% β-cyclodextrin; Veh) on every day of the sensory preconditioning training. Rats were injected and returned to their home cage for 30 mins before being transferred to the behavioral training chamber for sensory preconditioning.

## Sensory preconditioning (SPC)

Sensory preconditioning (SPC) training began after the SA training and withdrawal period. The SPC procedure consisted of three stages, trained over 11 consecutive days, with drug treatment injections (Veh or D3a, 30 min pre-training) administered each day. Generally, rats received one session per day where each session contained 12 trials (variable inter-trial interval, M = 600s ± 300s), in which stimuli were presented non-contingently with or without reinforcers. The stimuli were four distinct 10s auditory cues, A and C (clicker and white noise, counterbalanced), B and D (tone and siren, counterbalanced). During conditioning, reinforcement entailed the delivery of 3 sucrose pellets within the 10s presentation of stimulus B, such that a pellet was delivered at 3, 6.5, and 10s. Immediately prior to preconditioning, rats were first shaped to receive food pellets from the magazine in a single session with 16 reinforcers (two sucrose pellets) delivered on a variable time schedule (*M* = 120s ± 60s).

1. Preconditioning: Rats received two days of preconditioning where two distinct S_1_-S_2_ stimulus pairs were presented: A->B and C->D. A stimulus pair consisted of a 10s auditory stimulus (clicker or white noise; A or C, counterbalanced) immediately followed by a second 10s auditory stimulus (tone or siren; B or D, counterbalanced). Each session consisted of a 6-trial block with one of the stimulus pairs (e.g. A->B), followed by a second block of the other stimulus pair (e.g. C->D). Block order was reversed on the second session, and fully counterbalanced across rats.
2. Conditioning: After 2 days of preconditioning, rats received 6 days of Pavlovian conditioning. Each session involved six reinforced trials of cue B (3 sucrose pellets delivered during cue presentation at 3, 6.5, and 9s), and six non-reinforced trials of cue D (no pellets delivered), presented in pseudo-random trial order.
3. Probe test: After conditioning, rats received 2 days of non-reinforced probe tests i.e. in extinction. On the first day, the probe test included a total of 6 trials of cue A and 6 trials of cue C, presented in alternating blocks of three trials of each stimulus (order counterbalanced across rats). On the second day, the probe test included a total of 6 trials of cue B (non-reinforced) and 6 trials of cue D, presented in pseudorandom order. The order of the probe test sessions was fixed for all rats (testing A/C on day 1, and B/D on day 2).

## Exclusion Criteria

Rats were excluded if they developed health issues during the experiment (Female n = 4, Male n = 5), if their catheter lost patency during SA training for cocaine and/or if they received fewer than 140 total infusions (cocaine or sucrose) over the 14 days of SA training (Female n = 7, male n = 4). Final numbers included in each cohort were: Cohort 1 (n = 5 Female, n = 6 Male), Cohort 2 (n = 4 Female, n = 6 Male), Cohort 3 (n = 6 Female, 5 Male), Cohort 4 (n = 12 Female, n = 12 Male), Cohort 5 (n = 13 Female, 15 Male). Final group numbers were: Suc_Veh N = 18 (n = 7 Female, n = 11 Male), Suc_D3a N = 15 (n = 6 Female, n = 9 Male), Coc_Veh N = 14 (n = 6 Female, n = 8 Male), Coc_D3a N = 17 (n = 10 Female, n = 7 Male).

The study aimed to include approximately n = 16 rats per group to achieve significant statistical power to detect the predicted treatment effect (based on pilot data, and published effect sizes using similar parameters [4,5]). This was achieved after training 5 cohorts. Analysis of the excluded animals did not reveal any systematic bias towards group membership or sex.

## Data analysis

***Self-Administration:*** During SA training, the primary measures were the total number of lever presses on the active lever, inactive lever, and the total number of infusions per session. To account for differences in the number of reinforcers available, data are expressed as a percentage of the maximum available number of reinforcers i.e. for each rat/session Percent Max Response = (Total Responses/60)*100 for all animals, except Sucrose SA rats in cohort 1 where Percent Max Response = (Total Responses/300)*100.

***Sensory Preconditioning:*** The primary response measure for SPC was the mean time spent in the food magazine during the auditory stimuli (CS), relative to baseline (PreCS, i.e. CS - PreCS). The baseline period was defined as the 10s immediately before stimulus presentation on each trial and was calculated separately for each corresponding stimulus/trial type. During the probe test, only the first 4 presentations of each stimulus were analyzed as this test was in extinction and responding rapidly declined during these sessions (however, this did not change the reported pattern of results).

Analysis of SA and each stage of SPC was conducted using a linear mixed effects model ANOVA that included the following factors where relevant: between-subjects factors of SA (Suc, Coc), Treatment (Veh, D3a), Sex (M, F), and within-subjects factors of Cue (AB, CD), Stimulus (S1, S2), and Session (1-6). Significant interaction effects were explained by follow up analyses of any significant differences in lower order interactions, effects, or simple effects. Rates of acquisition across sessions were analyzed using linear trend contrasts from a set of planned orthogonal polynomial contrasts. When relevant, a Holm-Šídák correction was used to determine the significance of simple effects to control the nominal family-wise error rate at α = .05.

All analyses were reported from full models that included Sex as a factor. Sex differences are reported fully in supplementary material; however, any observed significant sex differences were transient and were not observed by the end of conditioning in stage 2, or during the critical probe tests. Cohort number was included as a random effect in all analyses but was removed as it did not account for any variance in the data. For all linear mixed effects models, a maximal random effects structure was used for all repeated/within-subjects measures and systematically simplified to deal with non-convergence [6,7], and Satterthwaite approximation was used to calculate degrees of freedom.

For the probe test, two additional analyses were used to test how the SA and Treatment groups were solving the SPC task, and whether these solutions were similar between groups. These were (1) Correlations between S1 and S2 stimulus pairs, and (2) a behavioral similarity analysis.

***Correlations between S1 and S2 stimulus pairs:*** This analysis tested whether responding to cue A was predicted by B, and whether responding to cue C was predicted by D, and whether the strength of these relationships differed between each of the SA and Treatment groups. To achieve these simultaneous comparisons, a linear model was used to predict responding to S1 stimuli (A and C) with responding to S2 stimuli (B and D, respectively). The model included S1 as the outcome variable predicted by S2 x SA x Treatment x Cue x Sex (full factorial model, type III sums of square). S1 and S2 variables were continuous magazine duration (CS – PreCS) scores that were mean-centered within each cue. Sex did not contribute to the final model (main effects or interactions) and was dropped as a predictor from the final model. Follow up analyses to explain the higher order interaction were run separately for cue pairs AB and CD.

***Behavioral similarity analyses:***  To quantify the full pattern of behavior both between and within cues A-D, a behavioral similarity analysis was used (similar to representational similarity analyses [8]. To account for within cue behavior, responding was first separated into 5s time bins within each 10s cue. For each group, a Pearson cross-correlation matrix of all cues/time bins was calculated to generate a behavioral similarity matrix. These matrices were then compared to test the similarity of the behavioral solutions between groups. This behavioral similarity analysis was conducted with Spearman rank correlations on the lower triangle of each group cross-correlation matrix. a Holm-Šídák multiple comparison correction was used to control the family-wise error rate at α = .05.

***Statistical Software:*** All analyses were conducted in RStudio [9,10]. For specific analyses, the following packages were used: linear models - lm(), correlations - cor(), cor.test(), family-wise error rate correction - p.adjust (stats); Type III sums of squares for linear models, Anova() [car - 11]; Linear mixed effects models - mixed() [afex - 12]; Simple effects – emmeans() [emmeans - 13].

# Supplementary Analysis

## Supplementary analysis 1: Self-Administration

***SA Sex differences:*** There were significant sex differences in sucrose but not cocaine SA (SA x Sex, $F\left( 1,60.08 \right)=4.21$, $p=.045$; SA x Lever x Sex, $F\left( 1,60.07 \right)=3.30$, $p=.074$; SA x Lever x Session x Sex, $F\left( 13,112.30 \right)=1.96$, $p=.031$). These sex differences were restricted to the active lever (SA x Sex x Session linear trend: Active Lever $t\left( 264.63 \right)=2.90$, $p=.004$; but not on the inactive lever, SA x Sex x Session linear trend: Inactive Lever, $t\left( 264.63 \right)=-0.61$, $p=.540$), and only for sucrose SA (Sex x Session linear trend: Active Lever - Sucrose, $t\left( 263.89 \right)=3.21$, $p=.001$; Sex x Session linear trend: Active Lever - Cocaine, $t\left( 265.33 \right)=-0.91$, $p=.365$). Specifically, while both sexes increased responding on the active lever, the rate of increase was significantly lower in females than males (Male: Sucrose, Active Lever - significant positive linear trend over Session, $t\left( 264.16 \right)=9.89$, $p<.001$; Female: Sucrose, Active Lever - significant positive linear trend over Session, $t\left( 263.71 \right)=3.85$, $p<.001$).

## Supplementary analysis 2: Stage 1 - Preconditioning

***Sex differences:*** In general, there were no main effects or interactions with sex, except for a significant SA x Treatment x Cue x Sex interaction ($F\left( 1,56 \right)=5.51$, $p=.022$; all other p > .118). This reflected a sex difference in Suc_Veh group males that responded higher overall to the AB than the CD cues compared to Suc_Veh females, a pattern that was not significant in the other groups.

Specifically, this significant 4-way interaction reflected a significant 3-way SA x Cue x Sex interaction in the Veh ($F\left( 1,28 \right)=5.70$, $p=.024$) but not the D3a groups ($F\left( 1,28 \right)=0.45$, $p=.508$). In the Veh treatment groups, this significant 3-way interaction revealed a significant 2-way SA x Sex interaction in Sucrose but not Cocaine groups (Vehicle - Sucrose: SA x Sex, $t\left( 28 \right)=2.36$, $p=.026$; Vehicle - Cocaine: SA x Sex, $t\left( 28 \right)=-1.09$, $p=.284$), such that responding to Cue pair AB was greater than CD in Suc_Veh but not Coc_Veh females (Suc_Veh: Females - AB > CD, $t\left( 28 \right)=2.40$, $p=.023$, Coc_Veh: Females - AB vs CD, $t\left( 28 \right)=-0.42$, $p=.675$), but not males (Suc_Veh: Males - AB vs CD, $t\left( 28 \right)=-0.77$, $p=.447$, Coc_Veh: Males - AB vs CD, $t\left( 28 \right)=1.18$, $p=.248$).

## Supplementary analysis 3: Stage 2 - Conditioning

***Sex Differences:*** There was a significant 5-way SA x Treatment x Sex x Cue x Session interaction ($F\left( 5,560 \right)=3.15$, $p=.008$), which was decomposed using interaction contrasts where Session was coded as polynomial linear trend contrast to quantify the rate of increase or decrease in responding over sessions. This 5-way interaction reflected significant sex differences in acquisition over sessions to cue B in Suc_Veh rats (linear trend contrast over sessions, Suc_Veh: Cue B, significant Sex x Session, $t\left( 560 \right)=2.91$, $p=.004$, Suc_Veh: Cue D, Sex x Session, $t\left( 560 \right)=-0.23$, $p=.817$; all remaining Sex x Session interactions for each cue and group, p > .067). Specifically, there was a significant linear increase in responding over sessions to cue B in the males but not the females (Males, $t\left( 560 \right)=6.72$, $p<.001$; Females, $t\left( 560 \right)=1.64$, $p=.102$). This was supported by a significant 3-way Sex x Cue x Session interaction only in the Suc_Veh group ($t\left( 560 \right)=-2.22$, $p=.027$; but not significant in the other groups, p > .256), and a significant 4-way SA x Sex x Cue x Session interaction for the Vehicle but not D3a groups (Vehicle, $t\left( 560 \right)=-1.86$, $p=.063$; D3a, $t\left( 560 \right)=0.23$, $p=.818$). However, since responding to cue B during the conditioning stage conflates anticipation and consumption of the pellets, it is hard to interpret the exact nature of these sex differences.

## Supplementary analysis 4: Stage 3 - Probe Test

**Direct comparison of the effect of vehicle and VK4-116 treatment on the SPC effect in Cocaine SA rats:** We hypothesized that VK4-116 would treat the deficits in SPC caused by a history of cocaine use, and present statistical support for the conclusion that the SPC effect was disrupted in the Coc_Veh group, and significant SPC effect (A > C) in the Coc_D3a group. However, a direct statistical test of this hypothesis would be to test whether the size of the A-C SPC effect is greater in the Coc_D3a than the Coc_Veh groups i.e. a Treatment x Cue interaction for S1 stimuli in Cocaine SA groups. This interaction was in the predicted direction but failed to reach significance, (Coc_D3a: A-C) – (Coc_Veh: A-C): $\Delta M=1.41$, 95% CI $\left[ -0.06, 2.88 \right]$, $t\left( 27 \right)=1.97$, $p=.060$. Given the limitations of interpreting a non-significant null-hypothesis significance test, we conducted a Bayes Factor analysis [14] to determine how much confidence we should have in this result as evidence for a null result compared to our hypothesized effect [bayesplay R package; ,15]. The Bayes Factor calculated from this model was $BF_{10}=3.51$, indicating moderate evidence [16] in favor of the predicted interaction effect. We based the values for our predicted interaction effect size on our previous findings in Wied et al [5] (see below). Importantly, we obtained consistent Bayes Factors using a wide range of reasonable estimates of the predicted effect to rule out any in parameter estimation.

**Likelihood:** $X=1.41|\theta\sim Scaled$-$Shifted$-$t\left( \mu=\theta,\sigma=0.72,\nu=27 \right)$

**Alternative Prior:** $H_{1}:$ $\theta\sim Normal\left( \mu=1.9,\sigma=0.4 \right)$

**Null Prior:** $H_{0}:$ $\theta\sim Normal\left( \mu=0,\sigma=0.4 \right)$

# Supplementary Figures


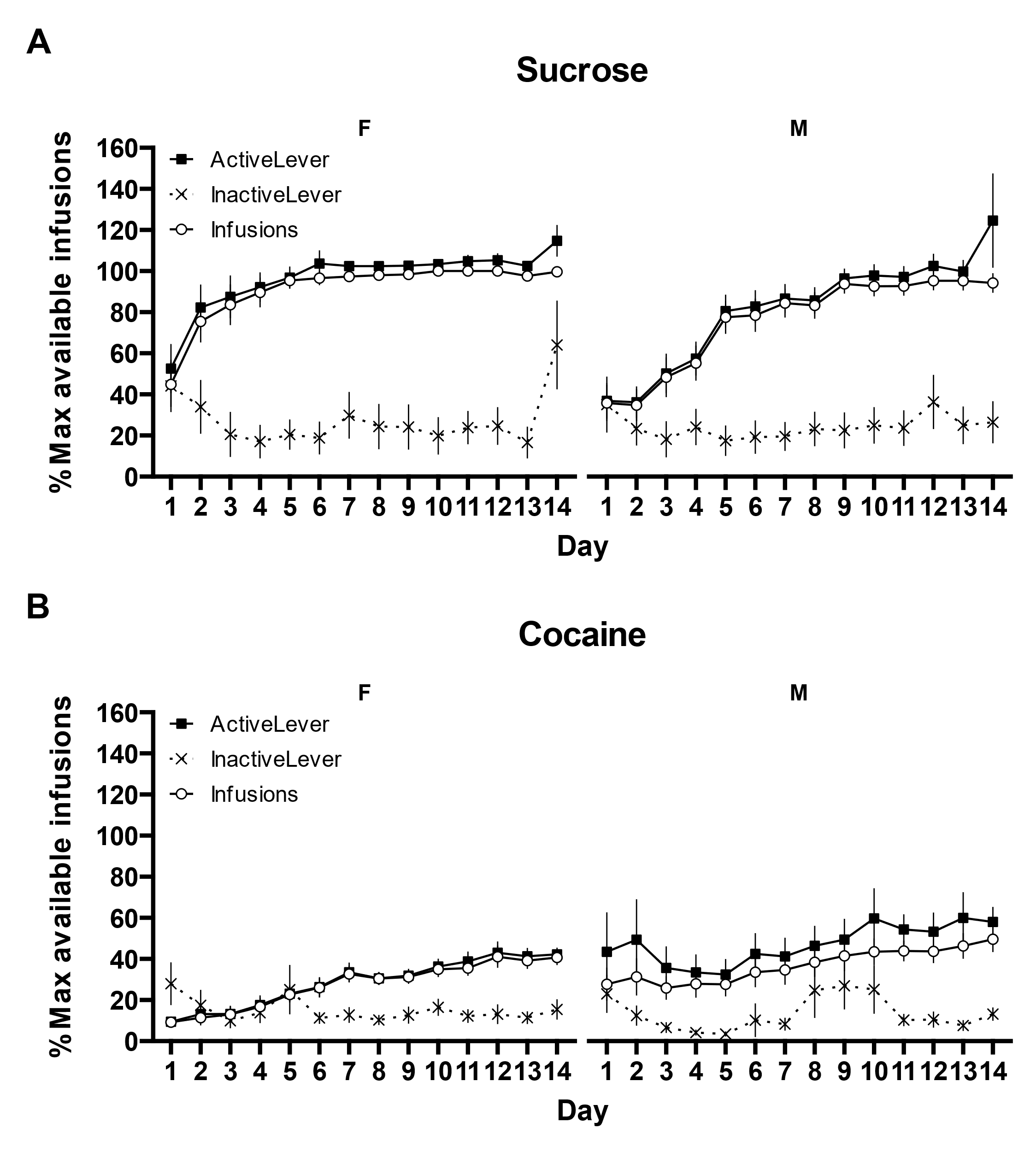
***Supplementary Figure 1.*** Sex differences in self-administration (SA) acquisition behavior. Rats in both the **(A)** sucrose and **(B)** cocaine groups successfully increased responding on the active lever, but not the inactive lever over 14 days of training. Data correspond to Figure 1 B & C, plotted separately for females (left) and males (right). The rate of response acquisition on the active lever was faster in males than females in the sucrose, but not the cocaine groups. Lever responses and reinforcer infusions are presented as a percentage of the maximum number of available infusions per session (max infusions was 60 for most animals; see methods for details). Error bars depict mean +/- SEM.


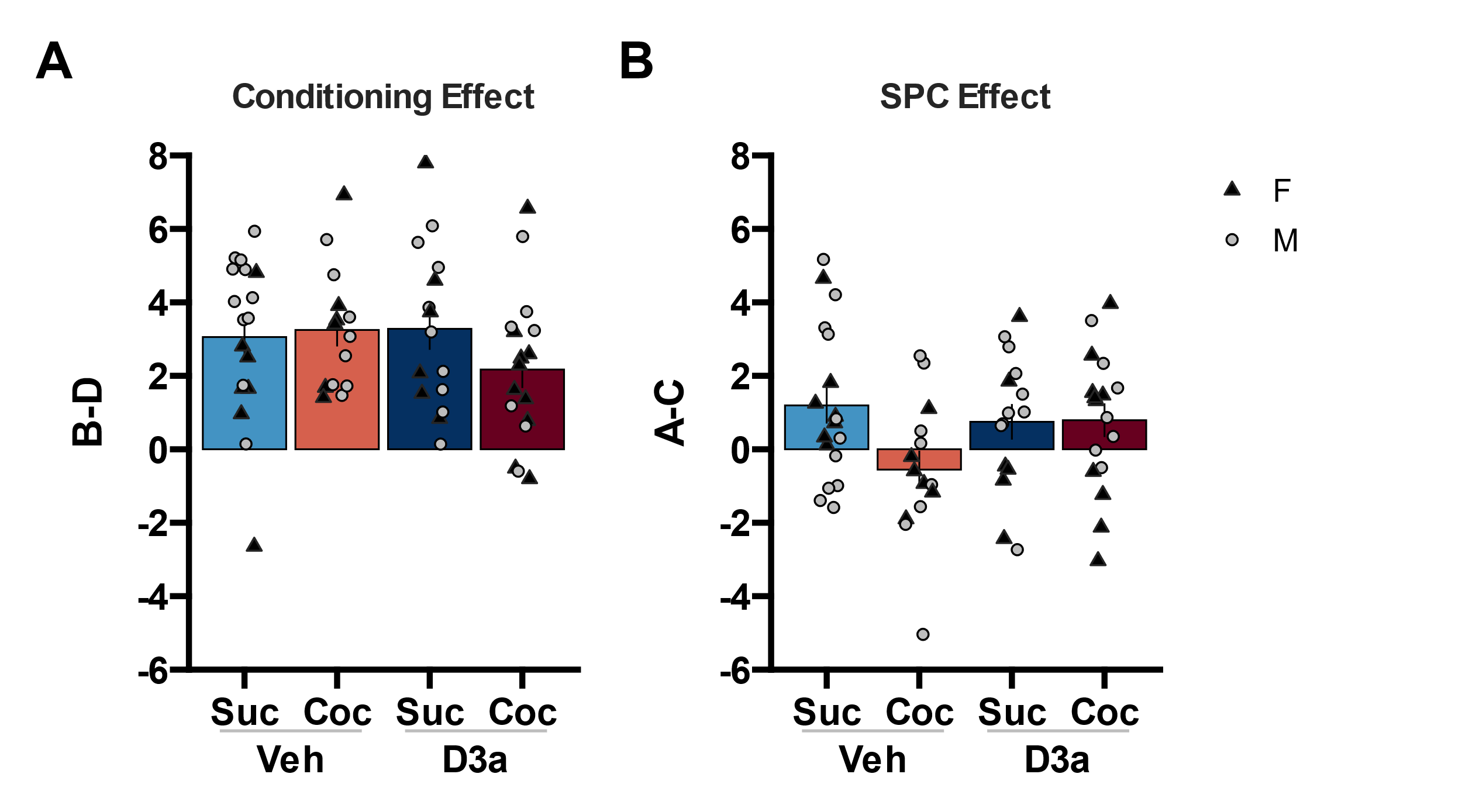


***Supplementary Figure 2.*** No sex differences in the conditioning and SPC effect during the SPC Probe Test. **(A)** The Conditioning effect: The difference in responding to cues B and D during the probe test provides an index of the conditioning effect such that scores above 0 reflect successful conditioning (data corresponds to Figure 2E). **(B)** The SPC effect: The difference in responding to cues A and C during the probe test provides an index of the SPC effect such that scores above 0 reflect successful SPC. Difference scores were calculated as the difference in discriminative responding to each cue from the Probe test in A-D (data corresponds to Figure 2F). The sex of individual subjects is represented as: Female = Circle, Male = Triangle. Error bars depict mean +/- SEM.


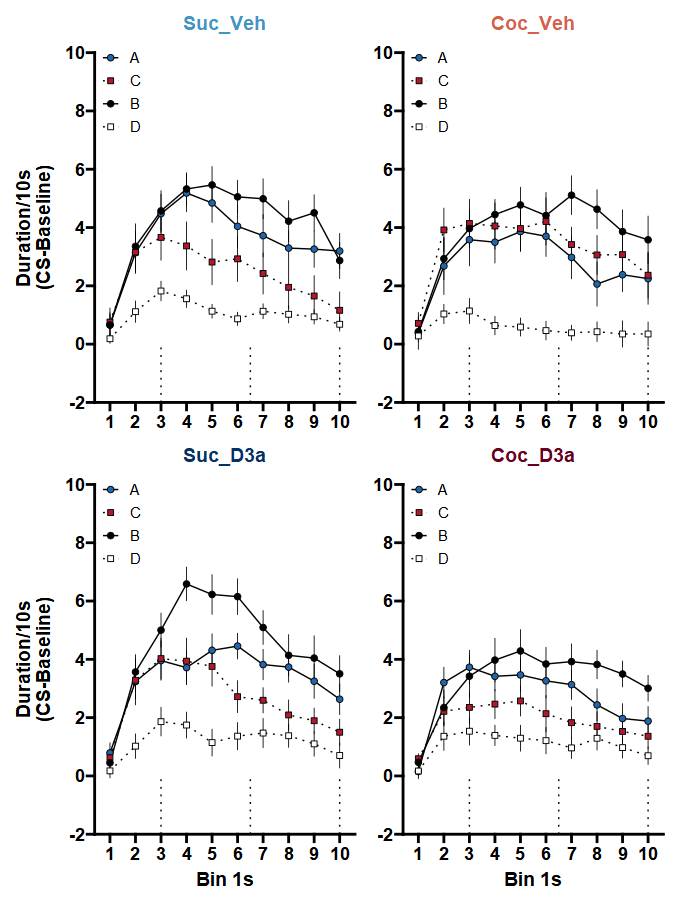
***Supplementary Figure 3.*** Within cue response patterns during the probe test for the Sucrose (left) or Cocaine (right) SA groups, and Vehicle (top) or D3-R antagonist (bottom) treatment groups. Responding in 1s time bins within each cue was calculated as the duration of time spent in the food cup during each time bin (as a rate per 10s), above the corresponding full 10s pre-CS baseline (mean +/- SEM). Dotted lines at 3, 6.5, and 10s indicate the time at which a pellet would have been delivered during reinforced presentations of cue B in stage 2 conditioning. The expected timing of pellet delivery is a within-cue response pattern that may reflect group specific solutions to the sensory preconditioning task.


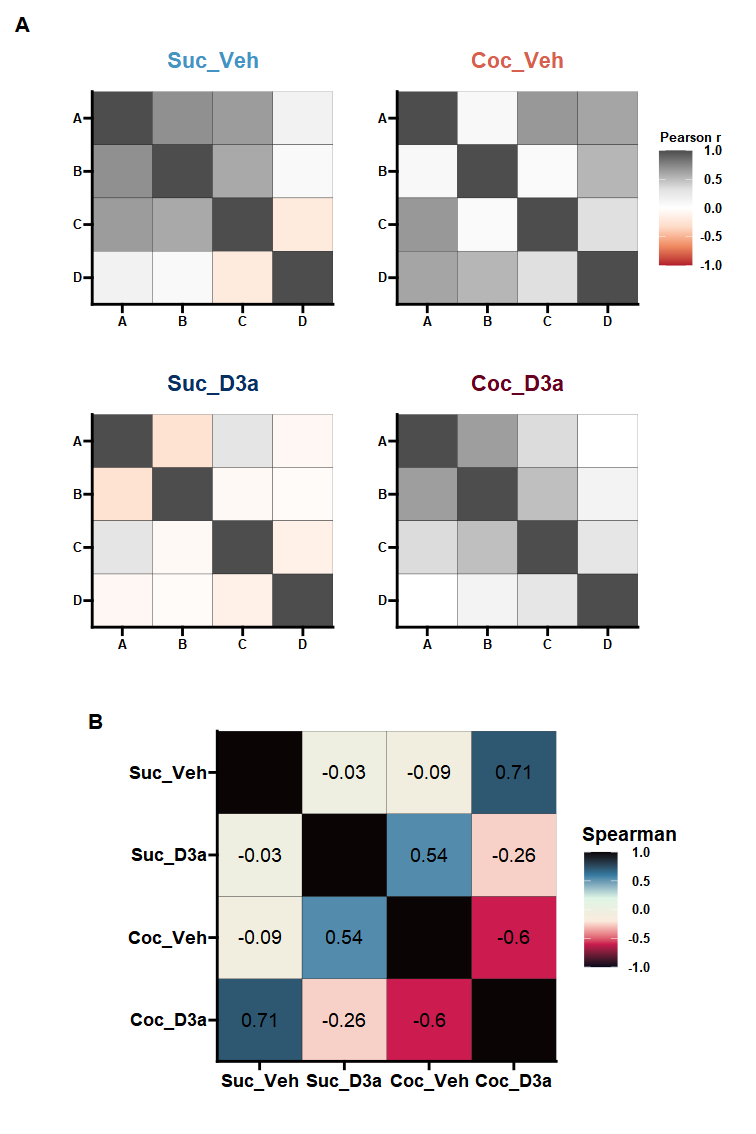
***Supplementary Figure 4.*** Behavioral similarity analysis of probe test responding between cues but ignoring within-cue response patterns (see Figure 4). **(A)** Behavioral similarity matrices for Sucrose (left) or Cocaine (right) SA groups, and Vehicle (top) or D3-R antagonist (bottom) treatment groups. For each group, the similarity of responding between cues was quantified by generating a cross-correlation matrix between all the cues. Color values are plotted to represent the correlation values (Pearson’s r), to provide a visual summary of the group specific pattern of response relationships. The behavioral similarity patterns were similar between the untreated control group (Suc_Veh) and the cocaine group treated with the D3-R antagonist (Coc_D3a), but different to the untreated cocaine group (Coc_Veh) and the control group treated with the D3-R antagonist (Suc_D3a). **(B)** *Behavioral similarity analysis:* A Spearman correlation was used to test the similarity between the group similarity matrices. Numbers (and corresponding color values) indicate the rank correlation (Spearman’s rho) between the lower diagonal of the cross-correlation matrices above.

# References

1. Mueller LE, Sharpe MJ, Stalnaker TA, Wikenheiser AM, Schoenbaum G. Prior cocaine use alters the normal evolution of information coding in striatal ensembles during value-guided decision-making. J Neurosci. 2021;41:342–353.

2. Kumar V, Bonifazi A, Ellenberger MP, Keck TM, Pommier E, Rais R, et al. Highly Selective Dopamine D 3 Receptor (D 3 R) Antagonists and Partial Agonists Based on Eticlopride and the D 3 R Crystal Structure: New Leads for Opioid Dependence Treatment. J Med Chem. 2016;59:7634–7650.

3. You Z-B, Bi G-H, Galaj E, Kumar V, Cao J, Gadiano A, et al. Dopamine D3R antagonist VK4-116 attenuates oxycodone self-administration and reinstatement without compromising its antinociceptive effects. Neuropsychopharmacology. 2019;44:1415–1424.

4. Jones JL, Esber GR, McDannald MA, Gruber AJ, Hernandez G, Mirenzi A, et al. Orbitofrontal cortex supports behavior and learning using inferred but not cached values. Science (80- ). 2012;338:953–956.

5. Wied HM, Jones JL, Cooch NK, Berg BA, Schoenbaum G. Disruption of model-based behavior and learning by cocaine self-administration in rats. Psychopharmacology (Berl). 2013;229:493–501.

6. Barr DJ, Levy R, Scheepers C, Tily HJ. Random effects structure for confirmatory hypothesis testing: Keep it maximal. J Mem Lang. 2013;68:255–278.

7. Meteyard L, Davies RAI. Best practice guidance for linear mixed-effects models in psychological science. J Mem Lang. 2020;112:104092.

8. Kriegeskorte N, Mur M, Bandettini P. Representational similarity analysis - connecting the branches of systems neuroscience. Front Syst Neurosci. 2008;2:1–28.

9. R Core Team. R: A Language and Environment for Statistical Computing. 2023.

10. Posit team. RStudio: Integrated Development Environment for R. 2023.

11. Fox J, Weisberg S. An R Companion to Applied Regression. Third. Thousand Oaks {CA}: Sage; 2019.

12. Singmann H, Bolker B, Westfall J, Aust F, Ben-Shachar MS. afex: Analysis of Factorial Experiments. 2023.

13. Lenth R V. emmeans: Estimated Marginal Means, aka Least-Squares Means. 2023.

14. Dienes Z. Using Bayes to get the most out of non-significant results. Front Psychol. 2014;5:1–17.

15. Colling LJ. bayesplay: The Bayes Factor Playground. 2023. 2023.

16. Aczel B, Palfi B, Szaszi B. Estimating the evidential value of significant results in psychological science. PLoS One. 2017;12:4–11.
